# The anti-inflammatory and immunomodulatory potential of braylin: Pharmacological properties and mechanisms by *in silico*, *in vitro* and *in vivo* approaches

**DOI:** 10.1371/journal.pone.0179174

**Published:** 2017-06-08

**Authors:** Renan Fernandes Espírito-Santo, Cassio Santana Meira, Rafael dos Santos Costa, Otávio Passos Souza Filho, Afranio Ferreira Evangelista, Gustavo Henrique Goulart Trossini, Glaucio Monteiro Ferreira, Eudes da Silva Velozo, Cristiane Flora Villarreal, Milena Botelho Pereira Soares

**Affiliations:** 1 Faculdade de Farmácia, Universidade Federal da Bahia, Salvador, Bahia, Brazil; 2 Instituto Gonçalo Moniz, Fundação Oswaldo Cruz (FIOCRUZ), Salvador, Bahia, Brazil; 3 Faculdade de Ciências Farmacêuticas, Universidade São Paulo, São Paulo, São Paulo, Brazil; 4 Centro de Biotecnologia e Terapia Celular, Hospital São Rafael, Salvador, Bahia, Brazil; Emory University, UNITED STATES

## Abstract

Braylin belongs to the group of natural coumarins, a group of compounds with a wide range of pharmacological properties. Here we characterized the pharmacological properties of braylin *in vitro*, *in silico* and *in vivo* in models of inflammatory/immune responses. In *in vitro* assays, braylin exhibited concentration-dependent suppressive activity on activated macrophages. Braylin (10–40 μM) reduced the production of nitrite, IL-1β, TNF-α and IL-6 by J774 cells or peritoneal exudate macrophages stimulated with LPS and IFN-γ. Molecular docking calculations suggested that braylin present an interaction pose to act as a glucocorticoid receptor ligand. Corroborating this idea, the inhibitory effect of braylin on macrophages was prevented by RU486, a glucocorticoid receptor antagonist. Furthermore, treatment with braylin strongly reduced the NF-κB-dependent transcriptional activity on RAW 264.7 cells. Using the complete Freund’s adjuvant (CFA)-induced paw inflammation model in mice, the pharmacological properties of braylin were demonstrated *in vivo*. Braylin (12.5–100 mg/kg) produced dose-related antinociceptive and antiedematogenic effects on CFA model. Braylin did not produce antinociception on the tail flick and hot plate tests in mice, suggesting that braylin-induced antinociception is not a centrally-mediated action. Braylin exhibited immunomodulatory properties on the CFA model, inhibiting the production of pro-inflammatory cytokines IL-1β, TNF-α and IL-6, while increased the anti-inflammatory cytokine TGF-β. Our results show, for the first time, anti-inflammatory, antinociceptive and immunomodulatory effects of braylin, which possibly act through the glucocorticoid receptor activation and by inhibition of the transcriptional activity of NF-κB. Because braylin is a phosphodiesterase-4 inhibitor, this coumarin could represent an ideal prototype of glucocorticoid receptor ligand, able to induce synergic immunomodulatory effects.

## Introduction

Immune-mediated disorders, such as rheumatoid arthritis, Crohn’s disease, asthma and ulcerative colitis, are a group of diseases associated with inflammatory pathogenetic mechanisms that involve an inappropriate or excessive immune response [1]. They affect approximately 5–7 percent of the population in Western countries [2,3]. The immune dysregulation causes inflammatory injury in various organs, resulting in morbidity, reduced quality of life and premature death [3]. The ideal drug to treat immune-mediated inflammatory disorders needs to stablish early control of inflammation, preventing the tissue damage, parallel to a favorable profile of adverse effects. Currently, the available anti-inflammatory drugs do not meet these requirements, often displaying more adverse effects than is acceptable, less therapeutic effects than desirable, or both.

Natural products have been considered a plentiful source in the search for new chemical entities that modulate the immune system with reduced adverse effects. Plant secondary metabolites are important for flavoring of food, resistance against pests and as drugs, including substances with immunosuppressive activity [4]. Coumarins, a group of plant-derived polyphenolic compounds, have attracted intense interest in recent years due to their diverse and potent pharmacological properties. The structural characteristic of coumarins depicts a framework consisting of fused benzene and α-pyrone ring systems [5]. This type of special benzopyrone structure enables its derivatives to exert noncovalent interactions with various active sites in organisms, such as enzymes and receptors, and thus display a wide range of biological activities [6]. In fact, these compounds have been considered to possess wide potential as medicinal drugs and have served as valuable leads for further design and synthesis of more active analogues.

**Fig 1:**
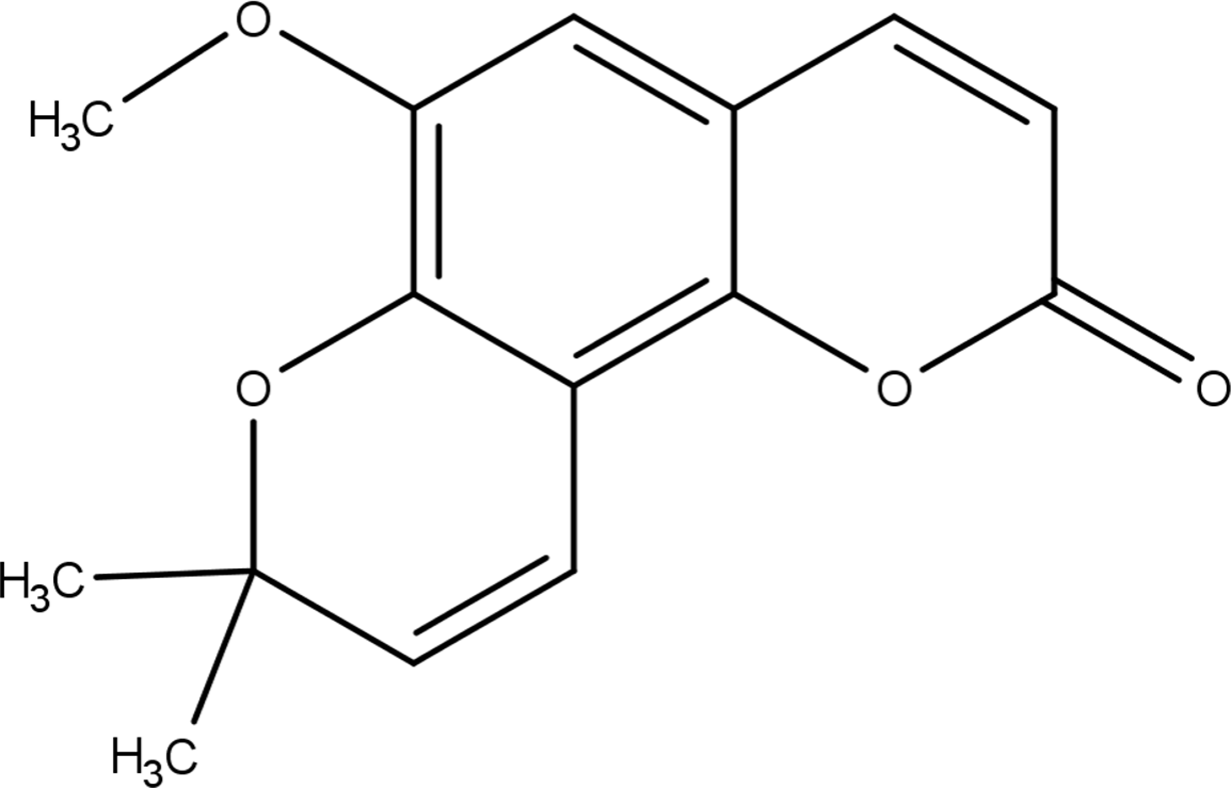
Chemical structure of braylin.

Among the multiple pharmacological properties of coumarins, their potent anti-inflammatory activity has been evidenced [7]. The anti-inflammatory properties of coumarin are associated with several mechanisms, including reduction of inflammatory molecules expression, inhibition of cyclooxygenase and lipoxygenase enzymes and inhibition of nuclear translocation of the transcription factor κB, NF-κB [7–10]. Braylin (Fig 1) is a coumarin first described in 1949, with limited data on their pharmacological properties already described [11]. Recently, Lin et al. showed that braylin presents potent phosphodiesterase-4 (PDE4) inhibitory activity [12]. Phosphodiesterases are enzymes that regulate the cellular levels of the second messengers cAMP and cGMP, by controlling their rates of degradation [13]. PDE4 is the predominant cyclic AMP degrading enzyme in a variety of inflammatory cells, and its inhibition elevates intracellular cAMP, which in turn down-regulates the inflammatory response [14,15]. Thus, this enzyme has been identified as a therapeutic target of high interest for immune-mediated inflammatory diseases [15–18]. Therefore, the present study was designed to evaluate whether braylin presents anti-inflammatory and immunomodulatory properties. Using *in vitro*, *in silico* and *in vivo* assays, we show here the pharmacological properties of braylin, including its possible mechanisms of action.

## Materials and Methods

### Extraction and isolation of braylin

Braylin was isolated from the roots of *Zanthoxylum tingoassuiba* A. St. Hil (Rutaceae) collected in August 2009 in Feira de Santana, Brazil, 12°12′52.9″ S, 38°52′44.1″ W. A voucher specimen (n°. 88005) has been identified and deposited at the Herbarium Alexandre Leal Costa (ALCB) of the Federal University of Bahia, Brazil. Braylin (837 mg) was isolated from the root bark (76.423 g) of *Zanthoxylum tingoassuiba* as a yellow amorphous solid and was identified by spectroscopic data comparison according to literature procedures [19]. ^1^H (500 MHz) and ^13^C (125 MHz) NMR spectra were acquired at room temperature on a VARIAN Inova-500 spectrometer, with CD_3_OD as solvent (S1 Table). The HPLC/ MS analysis was obtained with a HPLC Shimadzu 20A with Bruker micrO-TOF II spectrometer, using (N_2_) 10 eV for MS and 45 eV for MS/MS, in positive ionization mode with a Phenomenex Luna C_18_ column (4.6 × 250 mm, 5 μm particle size, 0.6 mL·min−1 oven at 35°C) (S1 Fig). Analytical HPLC analysis was carried out on a Shimadzu Prominence LC-6A instrument with Kromasil® C_18_ column (4.6 × 250 mm, 100A 5 μm particle size, 0.6 mL·min−1) and guard column (4.6 × 20mm, 5 μm particle size). All methods analyses were performed with isocratic flow of solvent A (MeOH) and solvent B (H_2_O) in proportion 50:50. HPLC eluates were monitored using UV detection at wavelengths of 254 nm. All solvents used were of analytical grade (Merck, Kenilworth, NJ, USA). The percent purity of braylin used in the pharmacological experiments carried out was greater than 98%, as determined by HPLC.

### Chemicals and drugs

Dexamethasone, antagonist of glucocorticoid receptor R486, complete Freund’s adjuvant (CFA), phosphate buffered saline (PBS), Tween 20, phenylmethylsulphonyl fluoride (PMSF), benzamethonium chloride, EDTA, aprotinin A, Dulbecco's Modified Eagle's Medium (DMEM), and 3,3´,5,5´- tetramethylbenzidine (TMB) were obtained from Sigma Chemical Company (St. Louis, MO, USA). Diazepam and morphine were obtained from Cristália (Itapira, SP, Brazil). Dexamethasone was dissolved in ethanol (10% in normal saline). Braylin was dissolved in 50% propylene glycol plus saline, and remaining substances were dissolved directly in saline. Drugs were administrated by intraperitoneal (ip) route 40 minutes before testing, and the control group only received vehicle.

### Peritoneal exudate macrophages cultures

Peritoneal exudate cells were obtained by washing, with cold saline, the peritoneal cavity of mice 5 days after injection of 3% thioglycolate in saline (1.5 mL per mouse). Cells were washed twice with DMEM, resuspended in DMEM supplemented with 10% fetal bovine serum (Cultilab, Campinas, Brazil) and 50 μg/mL of gentamycin (Novafarma, Anápolis, Brazil), and plated in 96-well tissue culture plates at 2 × 10^5^ cells per 0.2 mL per well. After 2 hours of incubation at 37°C, non-adherent cells were removed by two washes with DMEM. Macrophages were then submitted to the protocol of cytotoxicity, cytokine and nitric oxide determinations, as described below.

### Cytotoxicity to mammalian cells

To determine the cytotoxicity of braylin, murine macrophage-like cell line J774, kindly provided by Dr. Patricia S. T. Veras (Gonçalo Moniz Institute, Fiocruz/BA), Raw 264.7 Luc cells or peritoneal exudate macrophages were plated into 96-well plates at a cell density of 2 x 10^5^cells/well in Dulbecco’s modified Eagle medium (DMEM; Life Technologies, GIBCO-BRL, Gaithersburg, MD, USA) supplemented with 10% fetal bovine serum (FBS; GIBCO), and 50 μg/mL of gentamycin (Novafarma, Anápolis, GO, Brazil) and incubated for 2 hours at 37°C and 5% CO_2_. Braylin was added at four concentrations ranging from 10 to 80 μM in four replicates, and plates were incubated for 72 hours. Twenty μL/well of Alamar Blue (Invitrogen, Carlsbad, CA) was added to the plates during 12 hours. Colorimetric readings were performed at 570 and 600 nm. Gentian violet (Synth, São Paulo, Brazil) at 10 μM was used as positive control. Three independent experiments were performed.

### Measurement of cytokine and nitric oxide concentrations on macrophages

For cytokine and nitric oxide (NO) determinations, J774 cells or peritoneal exudate macrophages were seeded in 96-well tissue culture plates at 2 × 10^5^ cells/well in DMEM medium supplemented with 10% of FBS and 50 μg/mL of gentamycin for 2 hours at 37°C and 5% CO_2_. Cells were then stimulated with LPS (500 ng/mL, Sigma) and IFN-γ (5 ng/mL; Sigma) in the presence of braylin, vehicle or dexamethasone at different concentrations, and incubated at 37^°^C. Cell-free supernatants were collected 4 hours (for TNF-α measurement) and 24 hours (for IL-1β, IL-6 and nitrite quantification) and kept at -80^°^C. Cytokine concentrations in supernatants from macrophage cultures were determined by enzyme-linked immunosorbent assay (ELISA), using the DuoSet kit from R&D Systems (Minneapolis, MN, USA), according to the manufacturer’s instructions. For the antagonism assay, the glucocorticoid receptor antagonist RU486 was added in some cultures at a final concentration of 10 μM. Quantification of nitric oxide was done using the Griess method [20].

### NF-κB luciferase assay

The murine mouse leukemic monocyte macrophage cell line Raw 264.7 Luc cells bearing the pBIIX-luciferase (pBIIX-luc) targeting vector containing the firefly luciferase gene (luc) driven by two NF-kB binding sites from the kappa light chain enhancer in front of a minimal fos promoter [21] was kindly provided by Maria Célia Jamur (University of São Paulo, Ribeirão Preto, Brazil). Cells were cultured in RPMI medium (Sigma) supplemented with 20% FBS and 50 μg/mL of gentamycin at 37°C in a humidified environment containing 5% CO_2_. For luciferase reporter assays, 5 × 10^5^ cells/ml were pretreated with different concentrations of braylin (40, 20 or 10 μM) for 1 hour prior to stimulated with LPS (500 ng/mL) and IFN-γ (5 ng/mL) for 3 hours. Then each well was washed with cold-PBS and cells were incubated with TNT lysis buffer (200 mM Tris, pH 8.0, 200 mM NaCl, 1% Triton X-100) for 20 minutes at 4°C. The luciferase activity in the cell lysates was determined using the Luciferase Assay System (Promega, Madison, WI, USA). The samples were analyzed in a Globomax 20/20 luminometer (Promega). Data were then expressed as relative light units.

### Molecular docking

Molecular docking and scoring protocols were used as implemented in GOLD version 5.2 (CCDC, Cambridge, UK) [22] to investigate the possible ligand binding conformations within the glucocorticoid receptor (GR) binding pocket. X-ray crystallographic data for GR complexed with R486 group 2.3 A (PDB ID 1NHZ) used in the docking simulations were retrieved from the Protein Data Bank (PDB). The ligand and water molecules were removed from the binding pocket and hydrogen atoms were added in standard geometry using the Biopolymer module implemented in SYBYL 2.0 (Sybyl x 2.1. Tripos, 2010). The residues within the binding sites were manually checked for possible flipped orientation, protonation, and tautomeric states with Pymol 1.3 (Delano Scientific, San Carlos, USA) side-chain wizard script. The binding sites were defined as all the amino acid residues encompassed within a 10.0 A radius sphere centered on catalytic. Docking method was validated by redocking of the GR structure to crystal structure (PDB: 1NHZ) with R486.

### Animals

Experiments were performed on male Swiss Webster mice obtained from the Animal Facilities at the Instituto Gonçalo Moniz (FIOCRUZ; Salvador, Brazil). Animals (22–28 g) were housed in temperature-controlled rooms (22–25°C), under a 12:12 hours light-dark cycle, with access to water and food ad libitum until experimental initiation. All behavioral tests were performed between 8:00 a.m. and 5:00 p.m., and animals were only used once. Animal care and handling procedures were in strict accordance with the recommendations in the Guide for the Care and Use of Laboratory Animals of the National Institutes of Health and Brazilian College of Animal Experimentation. The protocol was approved by the Institutional Animal Care and Use Committee, Ethics Committee for Animal Experimentation of FIOCRUZ (CEUA/FIOCRUZ. Permit Number: L-IGM-015/2013). Every effort was made to minimize the number of animals used and any discomfort. Behavioral tests were performed without knowing to which experimental group each mouse belonged. Results shown are from two independent experiments performed.

### Inflammatory model

Mice were lightly anesthetized with halothane and received 20 μL of complete Freund’s adjuvant (CFA 1 mg/mL of heat killed *Mycobacterium tuberculosis* in 85% paraffin oil and 15% mannidemonoleate; Sigma) in the plantar region of the right hind paw, according to a previously reported method [23]. Inflammatory hyperalgesia, edema, and local cytokines levels were measured by von Frey filaments, plesthismometer and ELISA, respectively, as described below. Mice were injected with braylin (12.5 to 100 mg/kg), vehicle (50% propylene glycol in physiological saline; control group) or dexamethasone (2 mg/kg, reference drug) by ip route 40 minutes before CFA.

### Inflammatory hyperalgesia evaluation

The threshold to mechanical stimulation was measured with von Frey filaments (Stoelting, Chicago, IL, USA). In a quiet room, mice were placed in acrylic cages (12×10×17 cm) with wire grid floors, 30 minutes before the beginning of the test. This consisted of evoking a hind paw flexion reflex with one of a series of filaments with logarithmically incremental stiffness (0.0045–28.84 g). A positive response was characterized by the removal of the paw followed by clear flinching movements. A tilted mirror placed under the grid provided a clear view of the hind paws of the mice. An up-down method was used to record the threshold, which was represented as the filament weight (g) in which the animal responds in 50% of presentations [24].

### Plesthismometer test

The volume of each mouse paw was measured (mm^3^) with a plesthismometer (Ugo Basile, Comerio, Italy) before (Vo) and after (VT) the CFA injection, as described previously [23]. The amount of paw swelling was determined for each mouse and data were represented as paw volume variation (Δ, mm^3^).

### Cytokine measurement by ELISA

The paw levels of cytokines were determined as previously described [25]. Treatments were performed 40 minutes before the CFA injection. Skin tissues were removed from the paws 2, 4, 8 or 24 hours after CFA, in mice terminally anesthetized with halothane from each experimental group. Tissue proteins were extracted from 100 mg tissue/mL phosphate buffered saline (PBS) to which 0.4 M NaCl, 0.05% Tween 20 and protease inhibitors (0.1 mM PMSF, 0.1 mM benzethonium chloride, 10 mM EDTA, and 20 KI aprotinin A/100 ml) were added (Sigma). The samples were centrifuged for 10 minutes at 3000 g and the supernatant was frozen at -70°C for later quantification. Interleukin-1β (IL-1β), tumor necrosis factor α (TNF-α), interleukin-6 (IL-6), interleukin-13 (IL-13), interleukin-10 (IL-10) and transforming growth factor β (TGF-β) levels were estimated using commercially available immunoassay ELISA kits for mice (R&D System, Minneapolis, MN, USA), according to the manufacturer’s instructions. The results are expressed as picograms of cytokine per milligram of protein.

### Tail flick and hot plate tests

The tail flick test in mice was conducted as described elsewhere [26]. Before the experiment, each animal was habituated to the restraint cylinder for 20 minutes/day for 5 consecutive days. On the day of the experiment, mice were placed in the restraint cylinder and the tail tip (2 cm) was submersion in a water bath at 50 ± 0.5°C. The latency of the tail withdrawal reflex was measured in seconds. Each submersion was terminated after 16 seconds to minimize potential skin damage. Tail flick latency was measured before (baseline) and after treatments. The hot plate test in mice was conducted as described elsewhere, with minor modifications [27]. On the experiment day, mice were placed on the equipment (TECA Corporation, Chicago, IL, USA), which was maintained at 52 ± 0.5°C, and latencies to hind-paw licking or jumping (nociceptive thermal threshold) were recorded with a cut-off time of 16 s. The threshold was measured before (baseline) and after treatments.

### Motor function assay

To evaluate possible non-specific muscle-relaxant or sedative effects of braylin, mice were submitted to the rota-rod test, as previously described [26]. The rota-rod apparatus (Insight, Ribeirão Preto, SP, Brazil) consisted of a bar with a diameter of 3 cm, subdivided into five compartments. The bar rotated at a constant speed of 6 revolutions per minute. The animals were selected 24 hours previously by eliminating those mice that did not remain on the bar for two consecutive periods of 120 s. Animals were treated and 40 minutes afterwards were placed on a rotating rod. The resistance to falling was measured for up to 120 s. The results are expressed as the average time (s) the animals remained on the rota-rod in each group. Diazepam (10 mg/kg) was the reference drug.

### Statistical analysis

Data are presented as means ± standard error of the means (SEM) of measurements made on 6–9 animals in each group. Comparisons between three or more treatments were made using one-way ANOVA with Tukey’s post-hoc test, or for repeated measures, two-way ANOVA with Bonferroni’s post-hoc test, as appropriate. All data were analyzed using Prism 5 Computer Software (GraphPad, San Diego, CA, USA). Statistical differences were considered to be significant at *p* < 0.05.

## Results

**Fig 2:**
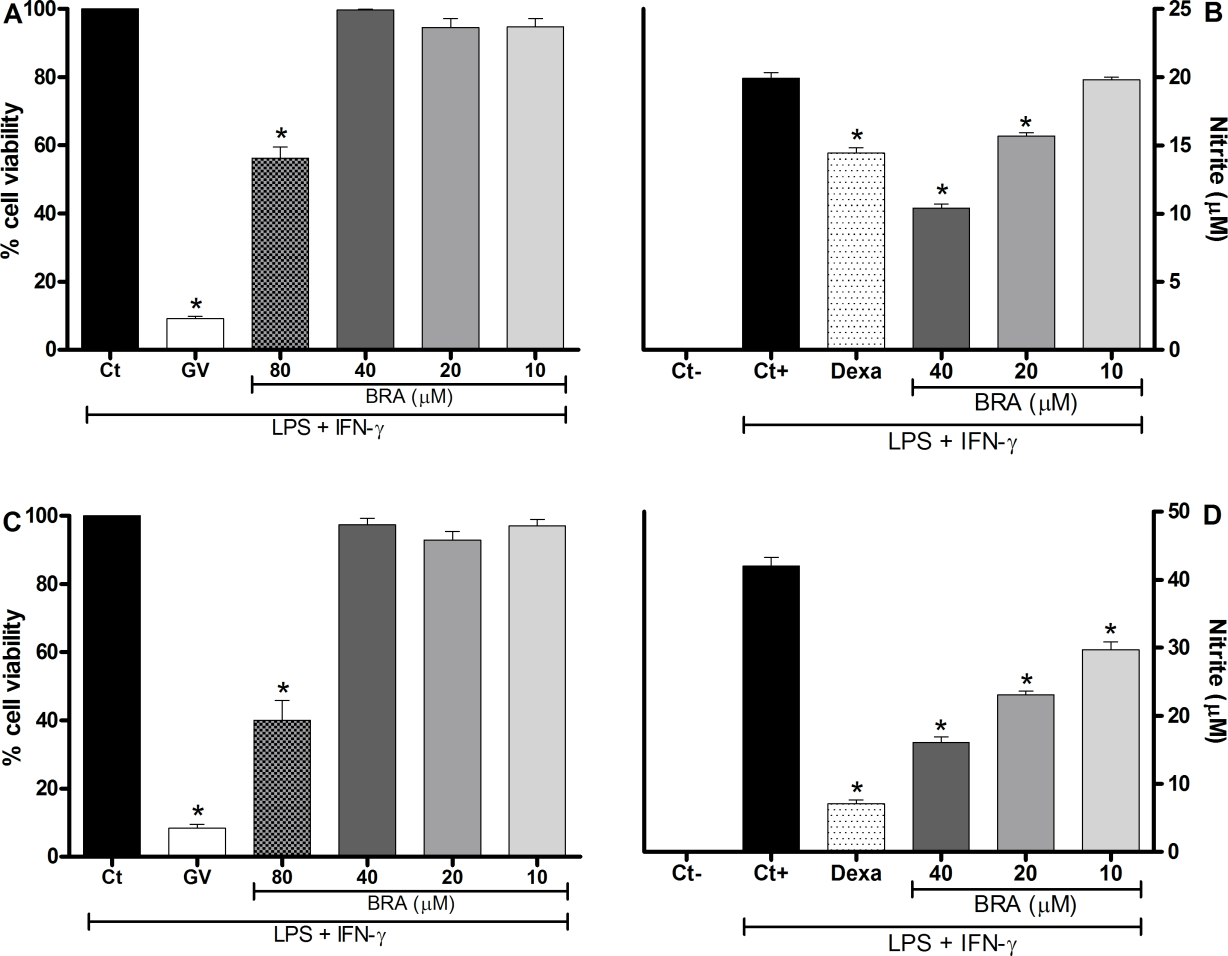
Cytotoxic effect of braylin and its modulation of nitric oxide production on macrophages. Panels A and C: J774 cells (A) or peritoneal exudate macrophages (C) were incubated with vehicle (50% propylene glycol in saline, Ct, control group) or different concentrations of braylin (BRA; 10, 20, 40 or 80 μM) for 72 hours and cell viability was determined by Alamar Blue assay. Gentian violet (GV) was used as positive control. Data are expressed as means ± SEM; *n* = 9 determinations per group. *Significantly different from the vehicle treated cultures (*p* < 0.05). ANOVA followed by Tukey´s multiple comparison test. Panels B and D: Concentrations of nitrite were determined in J774 macrophages (B) or peritoneal exudate macrophages (D) treated with vehicle (50% propylene glycol in saline, Ct+, control group), braylin (BRA; 10, 20 or 40 μM) or dexamethasone (Dexa; 40 μM) in the presence of LPS (500 ng/mL) + IFN-γ (5 ng/mL). Cell-free supernatants were collected 24 hours after treatments for nitrite quantification by the Griess method. Ct- shows concentrations of nitrite in unstimulated cells. Data are expressed as means ± SEM; *n* = 9 determinations per group. *Significantly different from the vehicle treated cultures stimulated with LPS + IFN-γ (*p*< 0.05). ANOVA followed by Tukey´s multiple comparison test.

Initially, the pharmacological effects of braylin were investigated on a set of *in vitro* assays. The effects of braylin on cell viability was determined by a colorimetric Alamar Blue assay 24 hours after treatment. As revealed in Fig 2, braylin at a concentration of 40 μM or lower did not induce cytotoxic effect on J774 cells or peritoneal exudate macrophages, stimulated with LPS and IFN-γ. Therefore, subsequent experiments were performed with braylin at 40 μM. The modulatory effects of braylin on macrophages were investigated through the quantification of the inflammatory mediators NO and cytokines, produced by activated macrophages. As shown in Fig 2B and 2D, braylin treatment reduced, in a concentration-dependent manner, the production of nitrite on macrophages stimulated with LPS and IFN-γ, suggesting a reduction of NO production. The inhibitory effect of braylin on J774 cells and peritoneal exudate macrophages was statistically significant until 20 and 10 μM, respectively. Dexamethasone at 40 μM reduced nitrite production.

**Fig 3:**
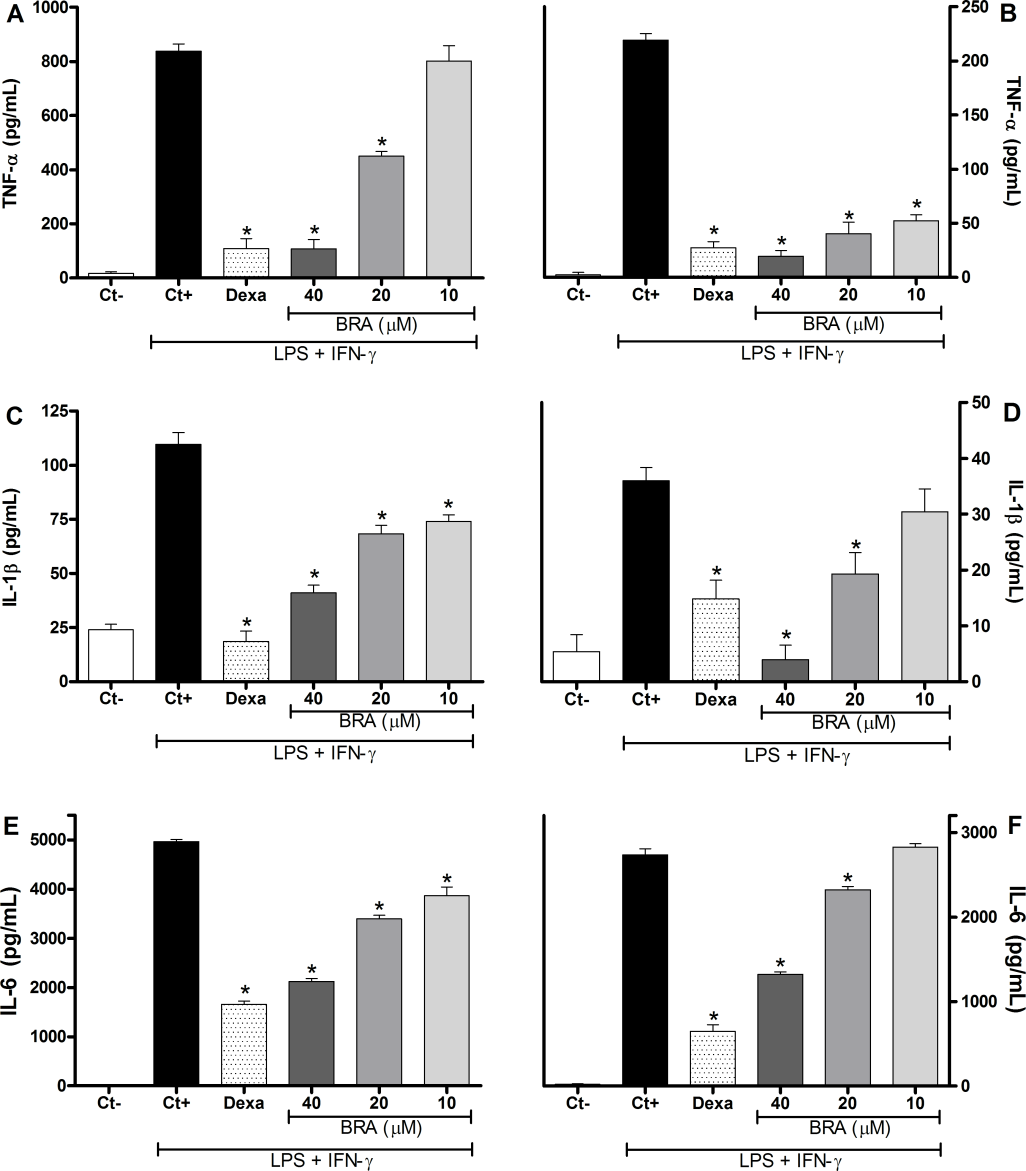
Effect of braylin on cytokine production by activated macrophages. Concentrations of TNF-α, IL-1β and IL-6 were determined in cultures of J774 macrophages (panels A, C and E) or peritoneal exudate macrophages (panels B, D and F) treated with vehicle (50% propylene glycol in saline, Ct+, control group), braylin (BRA; 10, 20 or 40 μM) or dexamethasone (Dexa; 40 μM) in the presence of LPS (500 ng/mL) plus IFN-γ (5 ng/mL). Cell-free supernatants were collected 4 hours (for TNF-α measurement) and 24 hours (for IL-1β and IL-6) after treatments for ELISA assay. Ct- shows cytokine concentrations in unstimulated cells. Data are expressed as means ± SEM; *n* = 10 determinations per group. *Significantly different from the vehicle treated cultures stimulated with LPS + IFN-γ (*p* < 0.05). ANOVA followed by Tukey´s multiple comparison test.

Fig 3 shows that braylin treatment was able to reduce, in a concentration-dependent manner, the production of TNF-α, IL-1β and IL-6 by macrophages stimulated with LPS and IFN-γ. Braylin reduced the production of TNF-α on J774 macrophages (Fig 3A) at concentrations of 20 and 40 μM. The inhibitory effect of braylin on the TNF-α production by peritoneal exudate macrophages was statistically significant at 10, 20 and 40 μM (Fig 3B). The braylin-induced reduction of IL-1β (Fig 3C) and IL-6 (Fig 3E) production on J774 macrophages was statistically significant at 10, 20 and 40 μM. In addition, braylin reduced the production of IL-1β (Fig 3D) and IL-6 (Fig 3F) on peritoneal exudate macrophages at 20 and 40 μM. Under the same conditions, dexamethasone (40 μM) caused a similar reduction of TNF-α, IL-1β and IL-6 production (Fig 3).

**Fig 4:**
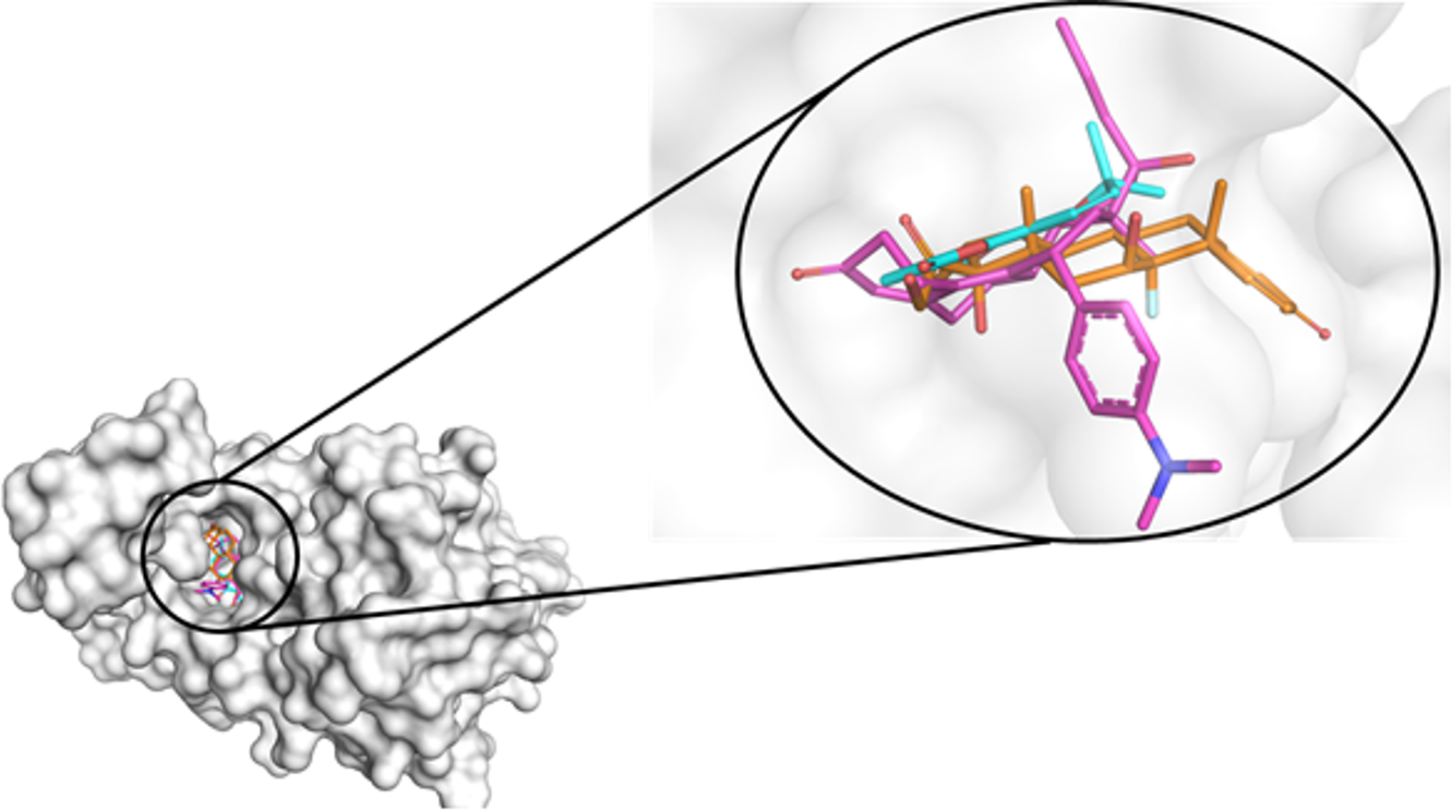
Best poses of the docking results to RU486 (pink), dexamethasone (orange) and braylin (cyan) superimposed in GR (pdb 1NHZ).

To assess the possible interactions of braylin in the binding pocket of glucocorticoid receptor (GR), docking studies were performed. The validation of the method showed a good superimposition between the crystal pose and the docked pose of RU486, a GR antagonist, suggesting that the docking method is sufficiently robust to determine the correct ligand poses in the active site of GR. The results were compared with docking poses of the antagonist RU486 and the agonist dexamethasone to suggest best fit interactions and supposed mechanism of action. The best poses obtained to RU486, braylin and dexamethasone in the glucocorticoid receptor (PDB: 1HNZ) are showed in Fig 4.

**Fig 5:**
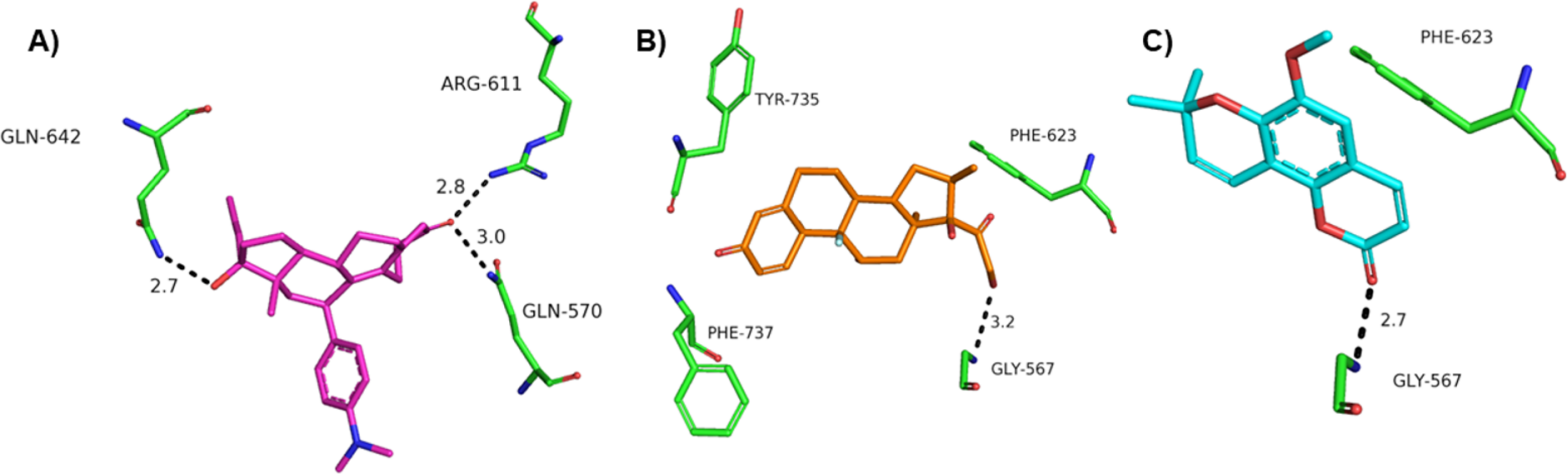
Docking solutions showing the main interactions for (A) RU486 (pink), (B) dexamethasone (orange) and (C) braylin (cyan) superimposed in GR (pdb 1NHZ).

Based on docking studies RU486, dexamethasone and braylin, presented interactions on the same site of the GR. When the occupancy was analyzed, RU486 showed interactions in two different subpockets, which are promoted by groups prop-1-yne and N,N-dimethylaniline. Dexamethasone presented a different subpocket interaction, promoted by ketone group and terminal ring (A) of the molecule. On other hand, braylin occupies only the active site of GR. The analyses of the interaction residues suggested by docking have shown that RU486 presents tree hydrogen bonds, with GLN 642, ARG 611 and GLN 570 (Fig 5A). For dexamethasone, the major interactions were observed in relation to dextrane group and two important hydrogen bonds with the carbonyl group of the TRY-735 and GLY-567 (Fig 5B). For braylin, the docking studies suggest the stabilization in the GR by hydrophobic interactions, hydrogen bond with GLY-567 like dexamethasone, and the PHE-623 promote a π- stack with aromatic ring (Fig 5C).

**Fig 6:**
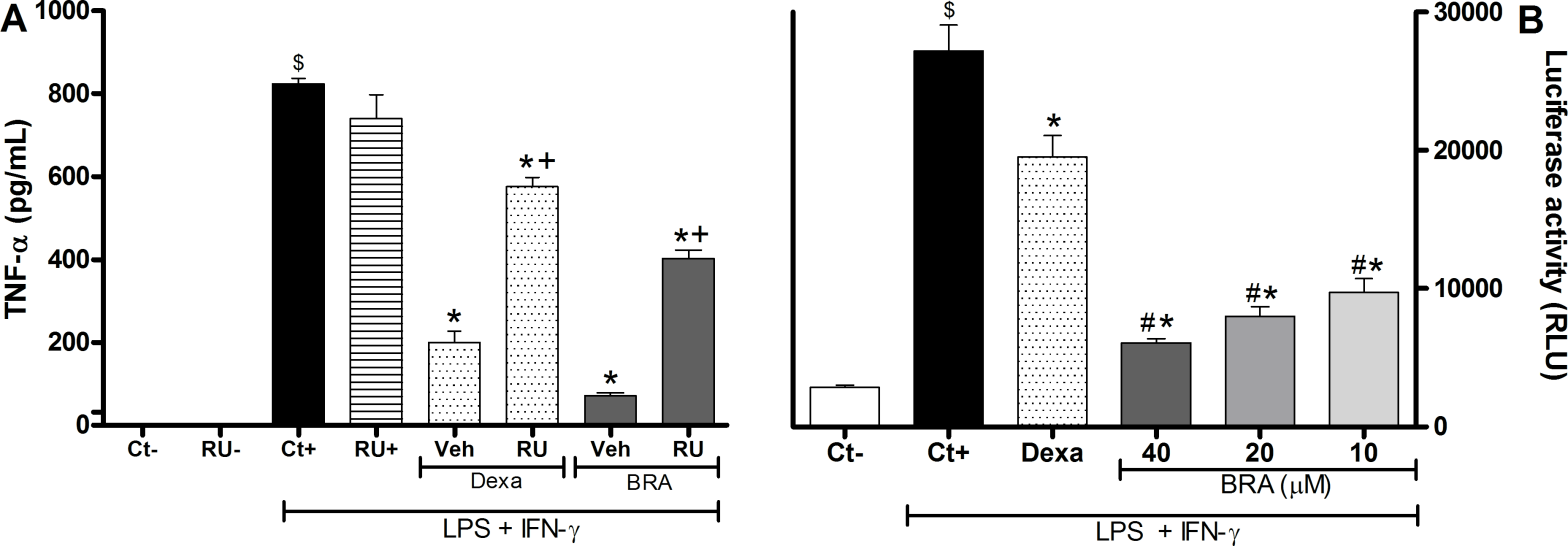
Involvement of glucocorticoid receptors and NF-κB dependent transcriptional activity in the immunomodulatory effect of braylin. Panel A shows data from glucocorticoid receptor antagonism assay. Concentrations of TNF-α were determined in J774 macrophages treated with vehicle (50% propylene glycol in saline, Ct+, control group), braylin (BRA, 40 μM), RU486 (GR antagonist, 10 μM) + braylin 40 μM, dexamethasone (Dexa; 40 μM) or RU486 (10 μM) + dexamethasone (40 μM) in the presence of LPS (500 ng/mL) and IFN-γ (5 ng/mL). Cell-free supernatants were collected 4 hours after treatments for TNF-α measurement by ELISA. Ct- and RU-show concentrations of TNF-α in unstimulated cells, treated with vehicle and RU486, respectively. Data are expressed as means ± SEM; *n* = 10 determinations per group. ^$^Significantly different from the vehicle treated cultures unstimulated (*p* < 0.05); *Significantly different from the vehicle treated cultures stimulated with LPS + IFN-γ (*p* < 0.05). ^+^Significantly different from the group untreated with antagonist (*p* < 0.05). Panel B shows the effect of braylin on the activation of NF-κB on RAW 264.7 Luc macrophages. Cells were pretreated with vehicle (50% propylene glycol in saline, Ct+, control group), braylin (BRA; 10, 20 or 40 μM) or dexamethasone (Dexa; 40 μM) for 1 hour prior to stimulated with LPS (500 ng/mL) and IFN-γ (5 ng/mL) for 3 hours. Ct- shows luciferase activity in unstimulated cells. Luciferase activity was measured in a luminometer. ^$^Significantly different from the vehicle treated cultures unstimulated (*p* < 0.05); *Significantly different from the vehicle treated cultures stimulated with LPS + IFN-γ (*p* < 0.05). ^#^Significantly different from the Dexa group (*p* < 0.05). ANOVA followed by Tukey´s multiple comparison test.

The possible antagonism of RU486 on the braylin-induced inhibitory effect in stimulated macrophage cultures was then evaluated. Addition of RU486 (10 μM) to J774 macrophage cultures stimulated with LPS and IFN-γ partially prevented the inhibitory effect of braylin (40 μM) on the TNF-α production (Fig 6A). As expected, in the presence of RU486, the inhibitory effect of dexamethasone (40 μM) on the TNF-α production by macrophages was reduced.

Next the NF-κB reporter system, in Raw 264.7 cells transfected with p-NF-κB-Luc reporter plasmid, was used to evaluate the effect of braylin on NF-κB activation. Initially, the effects of braylin on Raw 264.7 cell viability was determined. Braylin, at a concentration of 80 μM or lower, did not induce cytotoxic effect on Raw 264.7 cells 72 hours after treatment (S3 Fig). As revealed in Fig 6B, macrophage cultures stimulated with LPS and IFN-γ showed high levels of NF-κB dependent transcriptional activity. Treatment with braylin (10, 20 and 40 μM) dramatically reduced NF-κB dependent transcriptional activity when compared with vehicle-treated cultures. Treatment with dexamethasone (40 μM) also was able to reduce the NF-κB dependent transcriptional activity, but the effect of braylin, at all tested concentrations, was higher than that of dexamethasone.

**Fig 7:**
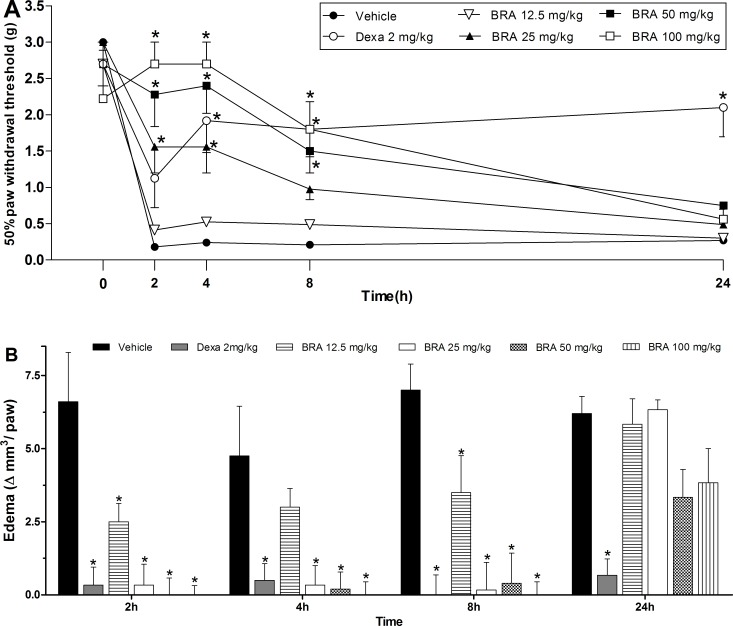
Effects of braylin on complete Freund’s adjuvant (CFA)-induced paw inflammation. Mice were injected with braylin (BRA; 12.5–100 mg/kg), vehicle (50% propylene glycol in saline; control group) or dexamethasone (Dexa; 2 mg/kg; reference drug) by ip route 40 minutes before CFA (injected at time zero). (A) Inflammatory hyperalgesia measured at 2, 4, 8 and 24 hours after the CFA stimulus. The mechanical nociceptive threshold (axis of ordinates) is represented as the filament weight (g) in which the animal responds in 50% of presentations. (B) Paw edema measured at 2, 4, 8 and 24 hours after CFA, represented as paw volume variation. Data are expressed as means ± SEM; *n* = 6 mice per group. * Significantly different from the control group (*p* < 0.05). Two-way ANOVA followed by the Bonferroni’s test.

Based on the reliable results from the *in vitro* assays, the potential of braylin as an immunomodulatory agent was next evaluated on the CFA-induced paw inflammation model. The effects of braylin on inflammatory hyperalgesia, paw edema and local levels of cytokines was assessed. Administration of braylin **(**25–100 mg/kg) by ip route, 40 minutes before CFA, significantly reduced inflammatory hyperalgesia at 2, 4 and 8 hours after the stimulus (Fig 7A). The pre-treatment with braylin (12–100 mg/kg, ip) significantly reduced paw edema 2, 4 and 8 hours post- stimulus (Fig 7B). Supporting data from braylin, vehicle treatment (50% propylene glycol in saline) yielded no activity, while the reference drug, dexamethasone (2 mg/kg), inhibited CFA-induced hyperalgesia and edema. Braylin had a greater efficacy than dexamethasone, considering both hyperalgesia (Fig 7A) and edema (Fig 7B) signs; however, its effects were short-lasting.

**Fig 8:**
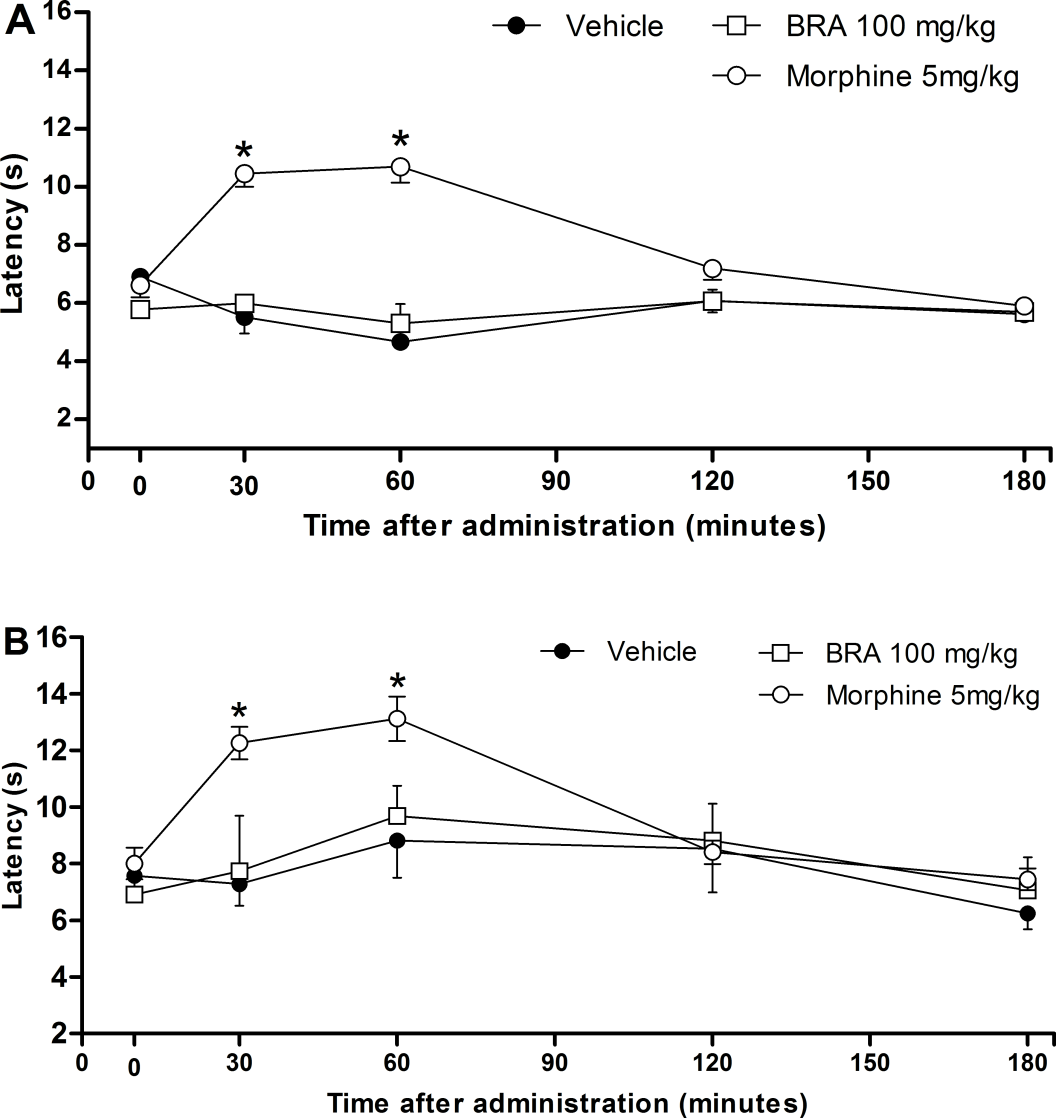
Effects of braylin on tail flick and hot plate tests in mice. Panels representing the latency in seconds in the tail flick (panel A) and hot plate (panel B) tests, after ip injection of braylin (BRA; 100 mg/kg), vehicle (50% propylene glycol in saline; control group) or morphine (5 mg/kg; reference drug). Data are reported as means ± SEM; *n* = 6 mice per group. * Significantly different from the control group (*p* < 0.05). Two-way ANOVA followed by the Bonferroni’s test.

The effects of braylin were also evaluated on the tail flick and hot plate tests, which mainly identify central analgesics. The ip administration of braylin (100 mg/kg) did not alter the latency time in the tail-flick (Fig 8A) and hot plate (Fig 8B) tests. The administration of morphine (5 mg/kg ip), the reference drug, resulted in a significant increase in the latency time at both, tail flick and hot plate tests (Fig 8). Moreover, relaxing or motor deficit effects were discarded, since administration of braylin (100 mg/kg, ip) did not affect the motor performance in mice on the rota-rod test (S4 Fig). As expected, diazepam (10 mg/kg ip), a central nervous system depressant used as standard drug, reduced the permanence time of mice on the rota-rod (S4 Fig).

**Fig 9:**
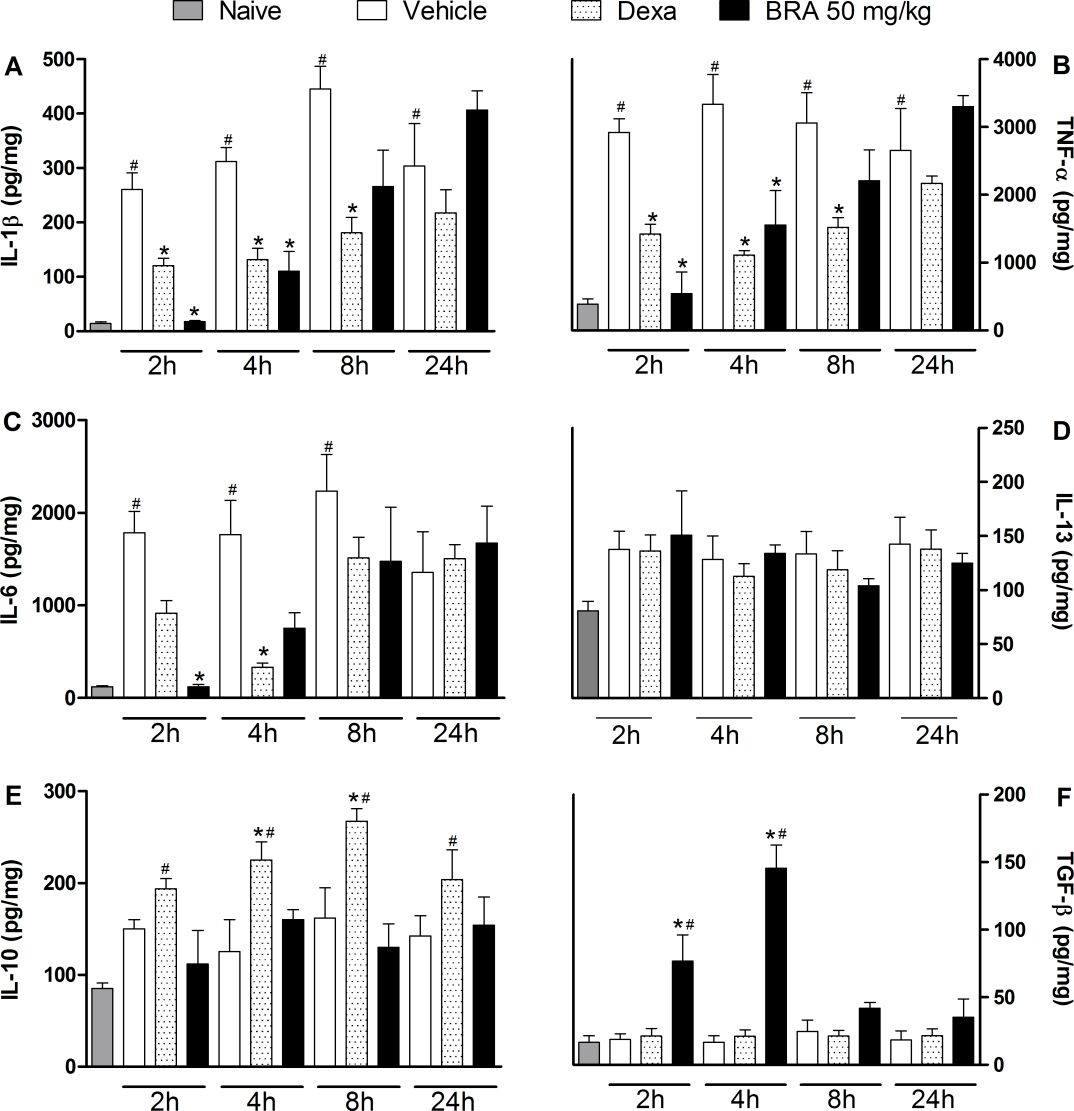
Effects of braylin on cytokines paw levels during CFA-induced inflammation. Mice were injected with braylin (BRA; 50 mg/kg), vehicle (50% propylene glycol in saline; control group) or dexamethasone (Dexa; 2 mg/kg; reference drug) by ip route 40 minutes before CFA (injected at time zero). The naïve group consists of mice that did not receive any experimental manipulation. Panels shows the paw levels of (A) interleukin-1β (IL-1β), (B) tumor necrosis factor-α (TNF-α), (C) interleukin-6 (IL-6), (D) interleukin-13 (IL-13), (E) interleukin-10 (IL-10) and (F) transforming growth factor-β (TGF-β), determined in skin tissues samples by ELISA, 3 hours after the CFA injection. The results are expressed as picograms of cytokine per milligram of protein. Data are expressed as means ± SEM; *n* = 6 mice per group. * Significantly different from the vehicle group in the same time (*p* < 0.05); ^#^ significantly different from the naive group (*p* < 0.05). ANOVA followed by Tukey´s multiple comparison test.

Considering the inhibitory effect of braylin on macrophage cells, its possible modulatory action on cytokine production during inflammation was evaluated. Data obtained by ELISA analyses shows that braylin (50 mg/kg) and dexamethasone (2 mg/kg) reduced the local levels of IL-1β (Fig 9A), TNF-α (Fig 9B) and IL-6 (Fig 9C) during CFA-induced paw inflammation. The inhibitory effects of braylin on TNF-α and IL-1β levels were statistically significant 2 and 4 hours after CFA, while on IL-6, a significant inhibition was seen 2 hours after stimulus. Dexamethasone reduced the IL-1β and TNF-α levels until 8 hours after stimulus, but the IL-6 level was reduced just until 4 hours. The modulatory effects of braylin on anti-inflammatory cytokines production were also investigated. Treatment with braylin (50 mg/kg) enhanced the paw levels of TGF-β (Fig 9F), while IL-13 (Fig 9D) and IL-10 (Fig 9E) were not modulated by this coumarin. Instead, dexamethasone enhanced IL-10, but not TGF-β or IL-13 concentrations.

## Discussion

The present study demonstrated, for the first time, the consistent anti-inflammatory and immunomodulatory properties of braylin. Braylin exhibited low cytotoxicity and consistent suppressive activities on macrophages cultures. Braylin acts, at least in part, through activation of GR, since the GR antagonist RU486 prevented the *in vitro* effects of braylin. Docking data corroborated with this hypothesis. In addition, using the NF-κB luciferase assay, the treatment with braylin dramatically reduced the NF-κB dependent transcriptional activity on macrophages. Systemic administration of braylin inhibited *in vivo* events related to inflammation, namely hyperalgesia and edema. Furthermore, this coumarin induced evident immunomodulatory property *in vivo* through the modulation of pro- and anti-inflammatory cytokines levels. These results describe the pharmacological properties of braylin, indicating this coumarin as a potential candidate to drug development.

Because macrophages play a central role in the immune regulation and inflammatory responses, the possible suppressive effect of braylin was initially evaluated on these cells. Activated macrophages release cytokines such as IL-1, IL-6, TNF-α, IL-10 and IL-18, that coordinate immune/inflammatory responses. They are activated by different signals, including inflammatory cytokines such as TNF-α, and deactivated by anti-inflammatory cytokines, such as TGF-β [28]. Activated macrophages are also capable of releasing high levels of NO, and this mediator has been implicated as a pro-inflammatory agent [29]. Braylin, at non-cytotoxic concentrations, reduced the production of nitrite, TNF-α, IL-1β and IL-6 by stimulated macrophages in a concentration-dependent manner, suggesting the anti-inflammatory and immunomodulatory potential of this coumarin.

To understand how braylin inhibits the production of inflammatory mediators by macrophages, the contribution of glucocorticoid receptor (GR) was first investigated by theoretical methods. Based on docking studies RU486, dexamethasone and braylin presented interactions on the same site of the GR. Analyzing the possible mode of interaction between the GR and RU486, dexamethasone and brailyn (S2 Fig), it was possible to verify that an important characteristic is the influence of the N,N-dimethylaniline group of RU486, which by steric hindrance displaces an alpha-helix, number 12, as previously observed [30–32]. In the mode of biding of dexamethasone and braylin, however, there is insufficient molecular volume to promote changes in conformation of the 12 alpha-helix due to the absence of groups that can confer this displacement. However, the mode of interaction with GR4, the specific receptor for dexamethasone, showed no withdrawal from this alpha-helix, thus suggesting a selectivity of dexamethasone and braylin by the GR receptor. The contribution of GR activation to the immunomodulatory effect of braylin was confirmed in an antagonism assay, in which RU486 partially prevented the inhibitory effect of braylin on stimulated macrophages.

Although glucocorticoids remain the most effective therapy for inflammatory and immune diseases, their use is associated with side effects and many patients with chronic diseases become resistant to glucocorticoids requiring higher doses [33]. Aiming to overcome this clinical problem, research has been focused on the development of more potent GR agonists or combination pharmacological strategies that target the GR, as well as other targets [34]. On the “combination therapy”, a second drug is added to potentiate the effects of the glucocorticoid. It has been demonstrated that the combined use with phosphodiesterase-4 inhibitors enhance the clinical efficacy of glucocorticoids, probably by elevating intracellular cAMP [35]. Importantly, braylin presents potent phosphodiesterase-4 inhibitory activity [12], in addition to a partially GR-dependent immunomodulatory effect of braylin demonstrated herein. Thus, according to the current goals of drug development, braylin can represent an ideal GR ligand prototype, able to cross-talk with other signaling pathways and inducing synergic immunomodulatory effects.

The GR activation mediates transactivation or transrepression of several genes involved with the reduction of inflammation and immune function [36]. Glucocorticoids induce their transcriptional effects by direct DNA binding of the GR or by binding to other transcription factors, such as NF-κB and AP-1, to repress their function [34]. In addition, an important signaling pathway used by Toll-like receptors in activated macrophages results in NF-κB activation. The genes that are expressed in response to NF-kB transcriptional activation encode several pro-inflammatory proteins, such as IL-1β, TNF-α, and inducible nitric oxide synthase [28]. Considering the inhibitory effects of braylin on these mediators, as well as the well-described crosstalk between NF-κB and glucocorticoid signaling, the effect of braylin on NF-κB activation was also evaluated.

We found here that braylin treatment was able to intensely reduce the transcriptional activity of NF-kB. Considering that NF-κB is a central regulator of inflammatory response, it is possible to propose that the mechanism of action of braylin, involved with its anti-inflammatory and immunomodulatory effects, is through the inhibition of the transcriptional activity of NF-κB. On the other hand, braylin possesses inhibitory activity on PDE_4_ [12], and PDE_4_ inhibitors present a broad range of anti-inflammatory activities in experimental and clinical conditions [16,37]. Whether or not the PDE_4_ inhibition contributes to the pharmacological effects of braylin presented here is still on investigation.

The potential of braylin as an immunomodulatory agent was also demonstrated *in vivo* using the CFA-induced paw inflammation model, a well-established experimental protocol for study of inflammation and anti-inflammatory drugs. CFA induces local release of mediators, such as cytokines and prostanoids, involved in the inflammatory signs, such as edema, hyperalgesia and vasodilation [38–42]. Importantly, systemic administration of braylin reduced the CFA-induced hyperalgesia with a greater efficacy than dexamethasone, considered the gold standard drug, showing an important and dose-related antinociceptive effect of braylin. The lack of effects in motor performance of mice on the rota-rod test reinforced the antinociceptive properties of braylin. The tail flick and hot plate tests, which mainly identify central-acting analgesics [43], indicated that the braylin-induced antinociception is not a centrally-mediated action, but likely an effect associated with anti-inflammatory properties.

Peripheral inflammation is associated with the local production of neuroactive inflammatory cytokines and growth factors. It has already been established that the local injection of CFA produces inflammatory hyperalgesia initiated by peripheral nociceptor activation and local release of mediators, such as IL-1β and TNF-α, which has a major role in the production of inflammatory pain hypersensitivity [38,44]. In addition to the inhibitory effect of braylin on macrophage cells *in vitro*, we showed that braylin reduced the local levels of IL-1β, TNF-α and IL-6 during CFA-induced paw inflammation. Cytokines play an essential role in the development of inflammatory signs and symptoms, and the first cytokines described as participating in the development of inflammatory pain were IL-1β, TNF-α and IL-6 [39,45,46]. In addition, upon inflammatory stimulation, the activation of the cytokine pathways precedes the release of final mediators such as prostaglandins, which are involved with nociceptive sensitization, in addition to its ability to trigger acute inflammation producing vasodilatation, vascular permeability and edema [46–51]. Considering the key role of IL-1β, TNF-α and IL-6 to the inflammatory response, it may be suggested that the antinociceptive and antiedematogenic effects of braylin are related to its ability to inhibit the release of inflammatory cytokines.

During the course of an inflammatory process, pro- and anti-inflammatory mediators are produced and the balance between these two signals determines the magnitude of the inflammatory response. In the present study, the antiedematogenic and antinociceptive effects of braylin were simultaneous to a substantial increase in the production of TGF-β. The TGF-β family members are cytokines that have been implicated in a broad range of biological functions including modulation of cell proliferation or cell differentiation, immunosuppression, tissue repair, and neuroprotection [52–54], and their immune functions are mostly anti-inflammatory. Chen *et al* demonstrated a pivotal role for TGF-β in the regulation of immune response leading to suppression of synovial inflammation and matrix destruction in streptococcal cell wall-induced erosive polyarthritis [55]. Systemic administration of TGF-β prevents the relapse of autoimmune encephalomyelitis [56]. In addition, TGF-β inhibits the proliferation of glial cells and induces anti-inflammatory and immunosuppressive effects on these cells [57,58]. Considering the above described properties of TGF-β, it is possible to propose that the braylin-induced pharmacological effects are mediated by this anti-inflammatory cytokine. In addition, under inflammatory conditions, TGF-β inhibits TNF-α production [59], corroborating the results showed here. In fact, beneficial effects of TGF-β on models of pain have been evidenced. TGF-β induces antinociceptive effect, inhibits the activation and proliferation of microglia and astrocytes and reduces the expression of pro-inflammatory cytokines involved with neuropathic pain maintenance [58,60].

## Conclusions

In conclusion, to the best of our knowledge, this is the first *in vivo* demonstration of pharmacological properties of braylin. Using *in vivo* and *in vitro* approach, antinociceptive, anti-inflammatory and immunomodulatory properties of braylin were demonstrated, likely linked to GR activation and its ability to induce inhibition of the transcriptional activity of NF-κB. Our results demonstrate a strong potential of braylin as a candidate drug for the treatment of immune-inflammatory diseases.

## Supporting information

S1 Table NMR data of braylin (CD3OD, ^1^H 500 MHz; ^13^C 125 MHz). (TIF)

S1 Fig HPLC/MS analysis of braylin (250 mm; 4.6 mm; 5 μm, flow 0.6 mL/min, 35°C). (TIF)

S2 Fig Interactions of (A) RU486 (pink), (B) dexamethasone (orange) and (C) braylin (cyan) with the GR. In A it is possible to see the steric steric hindrance displaces promoted by N,N-dimethylaniline group of the RU486 in the alpha-helix 12. Dexamethasone and brailyn did not induce changes in alpha-helix 12.
(TIFF)

S3 Fig Cytotoxic effect of braylin on RAW 264.7 Luc macrophages. (TIF)

S4 Fig Effects of braylin on motor function assessed by rota-rod test in mice. (TIF)
