# Supplementary figures and images for: The anti-inflammatory and immunomodulatory potential of braylin: Pharmacological properties and mechanisms by in silico, in vitro and in vivo approaches
Source: PLoS One. 2017 Jun 8;12(6):e0179174. doi: 10.1371/journal.pone.0179174 (PMC5464642; doi:10.1371/journal.pone.0179174)

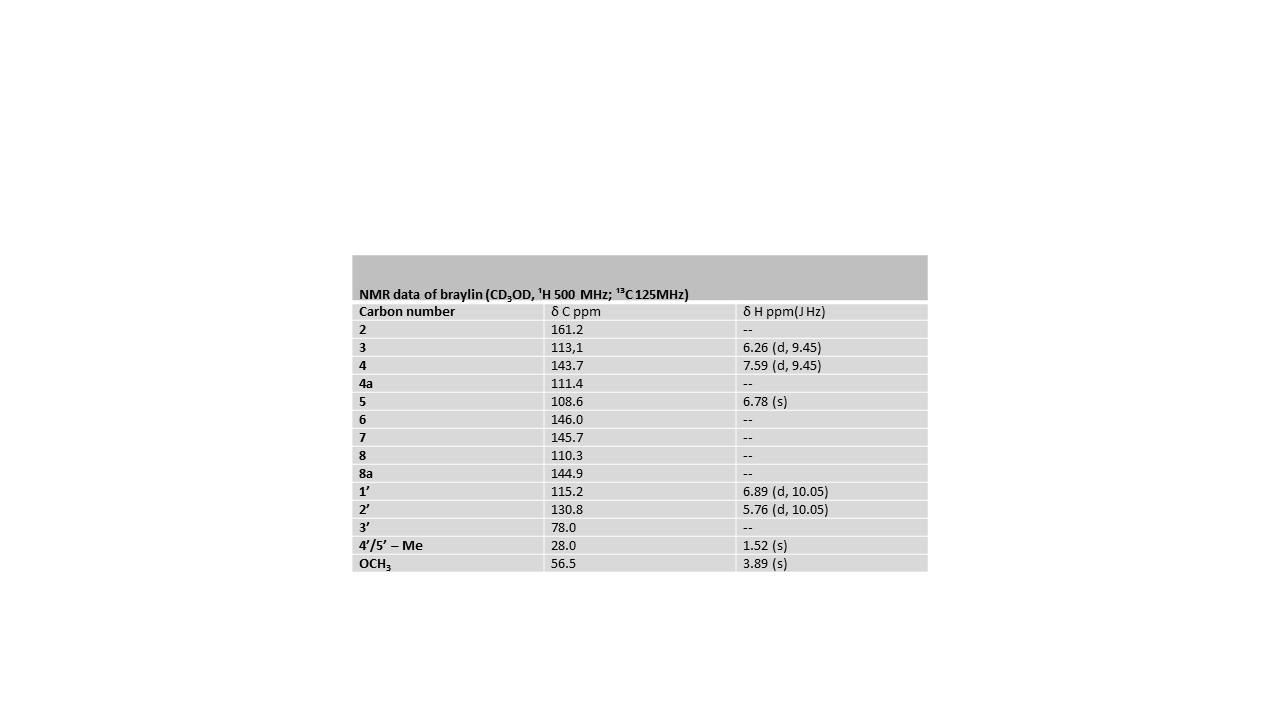

Supplement: S1 Table — (TIF) [file pone.0179174.s001.tif]

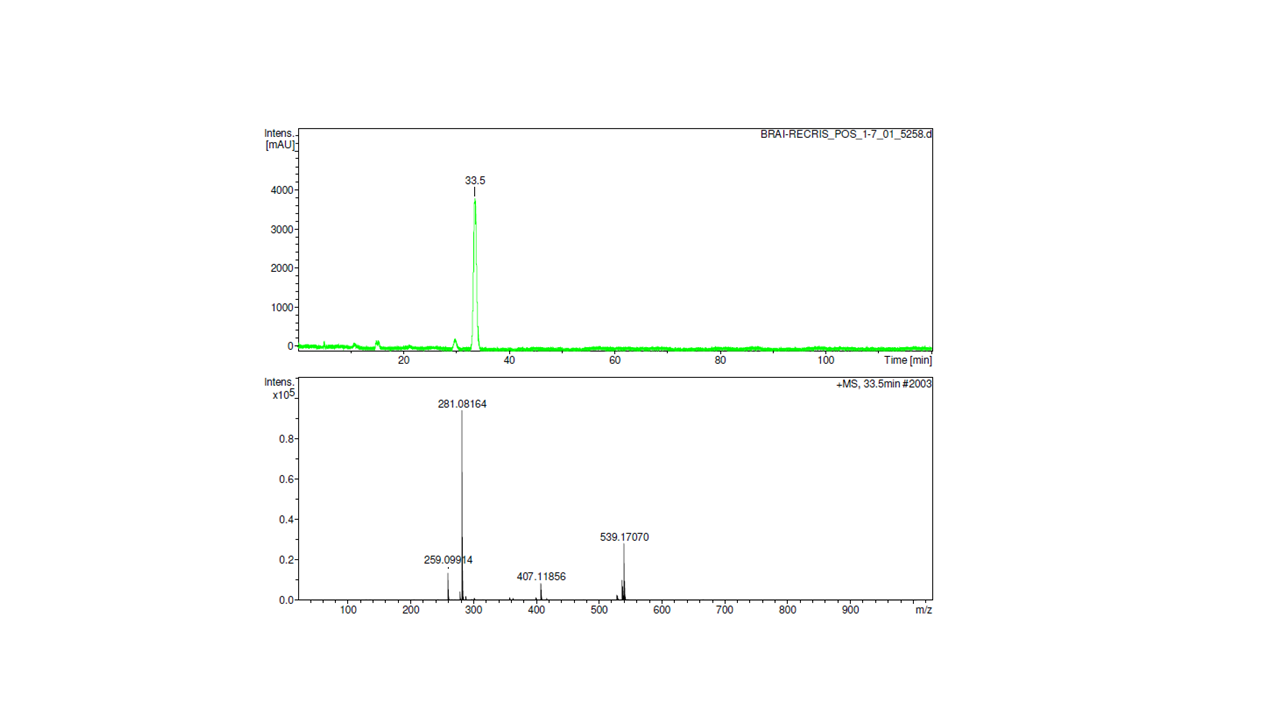

Supplement: S1 Fig — (TIF) [file pone.0179174.s002.tif]

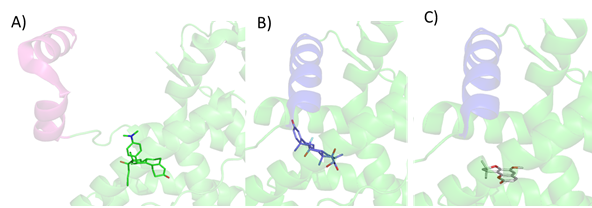

Supplement: S2 Fig — Interactions of (A) RU486 (pink), (B) dexamethasone (orange) and (C) braylin (cyan) with the GR. In A it is possible to see the steric steric hindrance displaces promoted by N,N-dimethylaniline group of the RU486 in the alpha-helix 12. Dexamethasone and brailyn did not induce changes in alpha-helix 12. (TIFF) [file pone.0179174.s003.tiff]

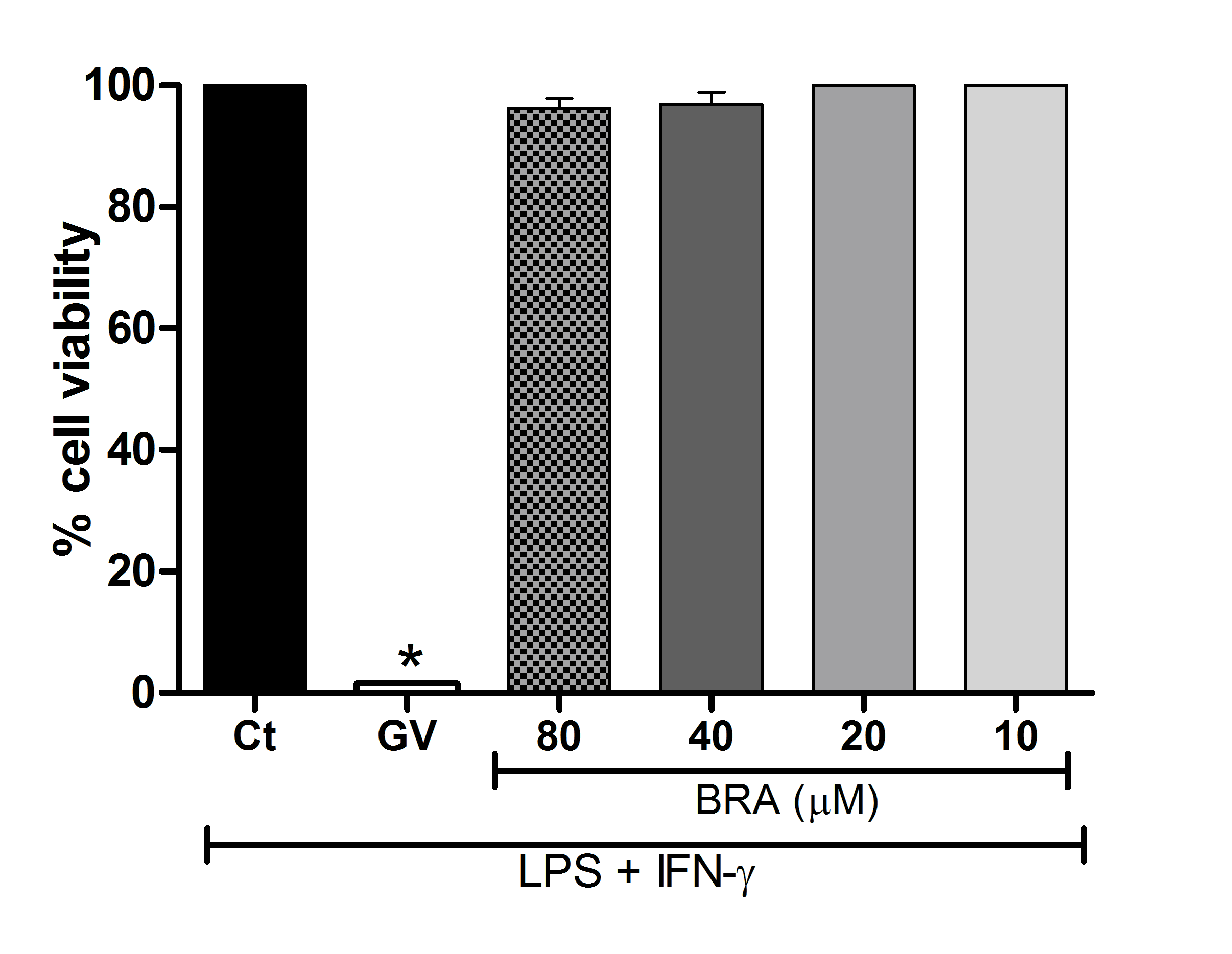

Supplement: S3 Fig — (TIF) [file pone.0179174.s004.tif]

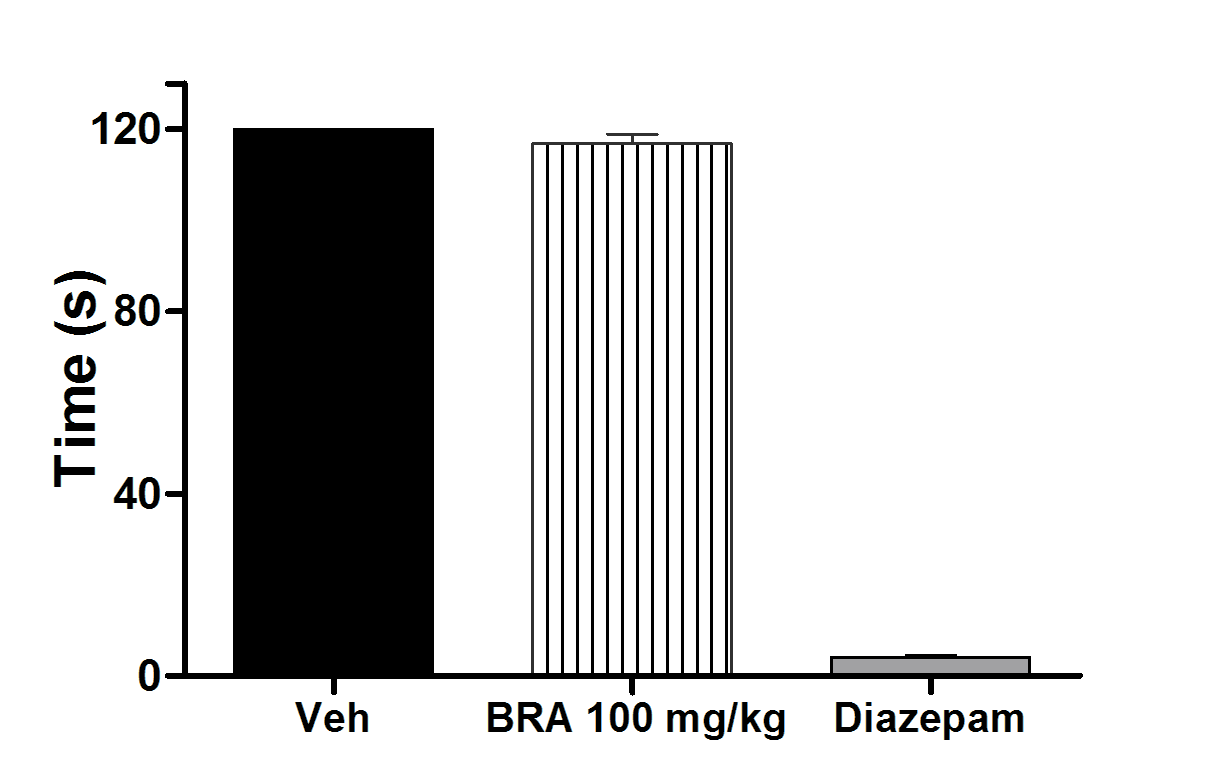

Supplement: S4 Fig — (TIF) [file pone.0179174.s005.tif]
